# Iron-Catalyzed Cross-Dehydrogenative Coupling

**DOI:** 10.3390/molecules30020250

**Published:** 2025-01-10

**Authors:** Haiyan Diao, Yujia Chen, Feng Liu

**Affiliations:** 1 Department of Chemistry, Fudan University, Shanghai 200438, China; 2 School of Chemistry and Chemical Engineering, Lanzhou Jiaotong University, Lanzhou 730070, China

**Keywords:** iron catalysis, cross-dehydrogenative coupling, C-H bond activation, green synthesis, organic transformations

## Abstract

This review highlights significant advances in iron-catalyzed cross-dehydrogenative coupling (CDC), a method pivotal for forming carbon-carbon (C-C) bonds directly from C-H bonds. This technique uses iron—a naturally abundant, inexpensive, and environmentally benign transition metal—as a catalyst to facilitate the coupling of two unfunctionalized C-H bonds. This method stands out for avoiding pre-functionalized substrates, reducing both waste and cost in organic synthesis. The discussion includes a variety of CDC methodologies involving combinations of C(sp^3^)-H with C(sp^3^)-H, C(sp^3^)-H with C(sp^2^)-H, and C(sp^3^)-H with C(sp)-H bonds. These methods have been successfully applied in synthesizing complex molecules and pharmaceuticals, highlighting the versatility and efficiency of iron catalysis.

## 1. Introduction

**Scheme 1:**
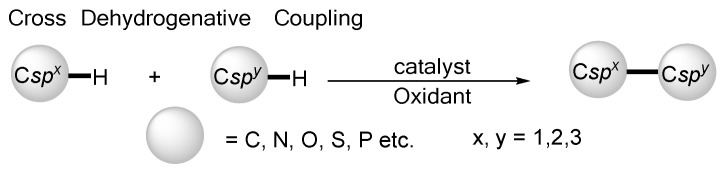
Cross-dehydrogenative coupling.

The formation of carbon-carbon (C-C) bonds remains one of the hottest areas of research in organic chemistry, crucial to synthesizing complex organic molecules from simpler ones. The cross-dehydrogenative coupling (CDC) of two unmodified C–H bonds is one of the most attractive and fundamental strategies for constructing C–C bonds (Scheme 1). This concept involves directly breaking two C-H bonds under the influence of an oxidant to form a carbon-carbon (C-C) bond [1]. This reaction type avoids the pre- and post-functionalization of substrates, generally yielding byproducts related only to the formation of H_2_ and H_2_O. It represents a highly effective atomic pathway, enabling shorter synthetic schemes and more abundant starting materials, thus enhancing synthetic efficiency and reducing reaction costs. Recently, this strategy has been widely applied to construct various important molecular skeletons and is considered feasible and efficient [2,3,4,5,6,7,8,9,10,11].

Iron is an abundant, cost-effective, non-toxic transition metal [12,13,14]. It not only exhibits properties expected of transition metals but also possesses characteristics of Lewis acids, such as promoting the formation of carbocation intermediates [15]; its non-toxicity is especially significant in pharmaceuticals, the food industry, agriculture, and cosmetics synthesis [16]. Moreover, iron complexes are present in biological systems [17], are essential nutrients, and participate in vital metabolic processes, such as those involving the cytochrome P450 enzymes [18]; iron’s variable spin states contribute to its chemical reactivity. These advantages make iron salts attractive catalysts or reagents in chemical transformations, considered ideal materials for developing catalysts [19]. In this review, we aim to provide an overview of Fe-catalyzed CDC reactions involving sp^3^ C-H bonds, including sp^3^-sp^3^, sp^3^-sp^2^, and sp^3^-sp C-C couplings, offering what we believe to be the first dedicated review on this specific topic.

## 2. Iron-Catalyzed Cross-Dehydrogenative Coupling of C(sp^3^)-H with C(sp^3^)-H Bonds

### 2.1. CDC Involving α-Position to Carbonyl C(sp^3^)-H Bonds

Iron-catalyzed CDC reactions have achieved notable success in recent years [11,20,21,22,23,24,25,26,27], capable of activating inert C-H bonds to directly form C-C and C-X (X = heteroatom) bonds.

**Scheme 2:**
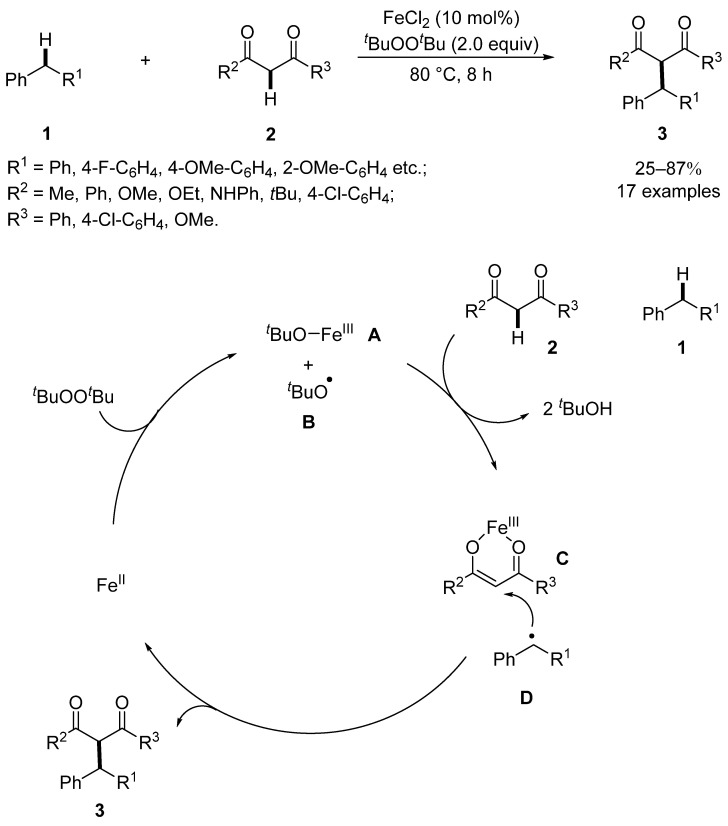
Selective CDC reaction of benzyl C-H bond catalyzed by FeCl_2_.

In 2007, Professor Li and his colleagues first reported a FeCl_2_-catalyzed selective CDC reaction between the benzyl C(sp^3^)-H bond **1** and the active methylene C-H bond of diketone compounds **2** (Scheme 2) [28]. This strategy applies to both cyclic and acyclic benzyl compounds, and the reaction exhibits good compatibility with aryl rings carrying electron-withdrawing or electron-donating groups and functionalized diphenylmethanes. The authors proposed a single electron transfer (SET) pathway where *^t^*BuO-O*^t^*Bu interacts with Fe(II) to yield Fe(III)–O*^t^*Bu species **A** and tert-butoxy radical **B**. Homolytic cleavage of the peroxy bond in *^t^*BuOO*^t^*Bu generates two tert-butoxy groups, capturing hydrogen from the benzyl substrate to form benzyl radical **D**, which then reacts with a diketone compound to form a chelated Fe-enolate complex intermediate **C**, finally forming the CDC product **3** through the attack of **D** on **C**, thus regenerating the Fe(II) species and completing the catalytic cycle.

**Scheme 3:**
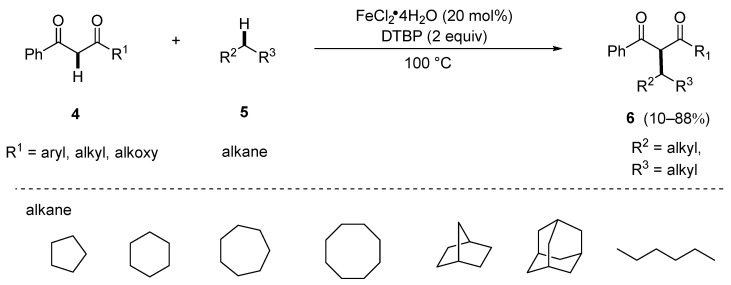
Direct alkylation of cycloalkane and activated methylene.

That same year, Li and his team also reported the CDC reaction of activated methylene with cycloalkanes. Using FeCl_2_·4H_2_O as the catalyst through the reaction of 1,3-dicarbonyl compounds **4** with equimolar amounts of alkanes **5**, the CDC reaction between C(sp^3^)-H and C(sp^3^)-H bonds was achieved (Scheme 3) [29]. Non-activated cycloalkanes, including five-, six-, seven-, and eight-membered rings, could be transformed into their corresponding products **6**. This mechanism also proceeded through a simple free radical addition reaction facilitated by the chelated Fe-enolate complex believed to be the active catalyst.

**Scheme 4:**
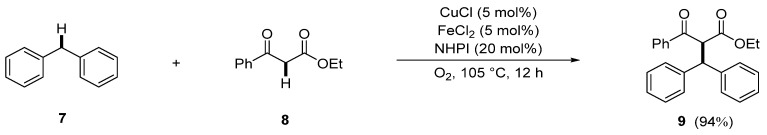
Oxygen acts as a terminal oxidant to catalyze the alkylation of benzyl C-H bonds.

Subsequently, Li further reported a bimetallic Cu(I)/Fe(II) catalytic system. Under catalytic amounts of N-hydroxyphthalimide (NHPI) and using O_2_ as the oxidant, a CDC reaction of 1,3-dicarbonyl compounds’ active methylene C(sp^3^)-H bonds **7** with benzyl C(sp^3^)-H bonds **8** was developed (Scheme 4) [30]. Additionally, less activated derivatives such as indene also underwent reaction under these conditions. If NHPI/O_2_/Cu(I) were responsible for forming the benzyl radical or benzyl alcohol, then the role of Fe(II) was to form the iron-enolate intermediate.

**Scheme 5:**
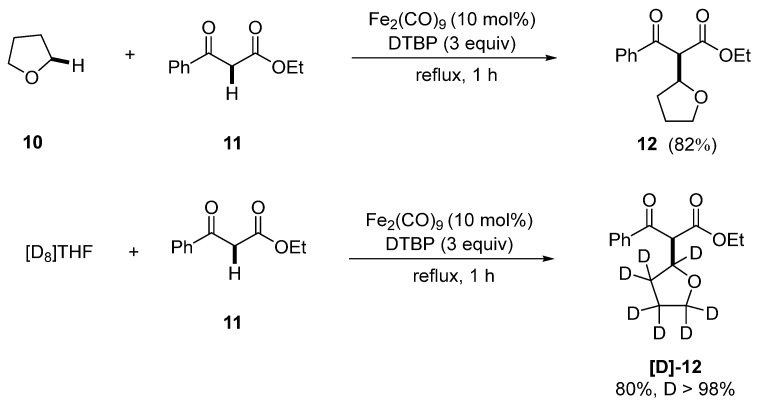
Iron catalyzes the activation of C-H bonds adjacent to heteroatoms.

Professor Li Zhiping and his colleagues reported in 2008 the iron-catalyzed CDC reaction of carbonyl-adjacent active methylene C(sp^3^)-H bonds **11** with heteroatom-adjacent C(sp^3^)-H bonds **12** (Scheme 5) [31]. Various cyclic and acyclic ether derivatives reacted with phenacyl acetate to yield the corresponding mixtures of non-enantiomeric products. Additionally, tetrahydrothiophene and N,N-dimethylaniline were suitable substrates. Isotope effects indicated that the rate-determining step of the reaction was the C-H bond cleavage, although the detailed mechanism remains unclear. It can be assumed that no iron-carbene complex is formed during the catalytic cycle.

**Scheme 6:**
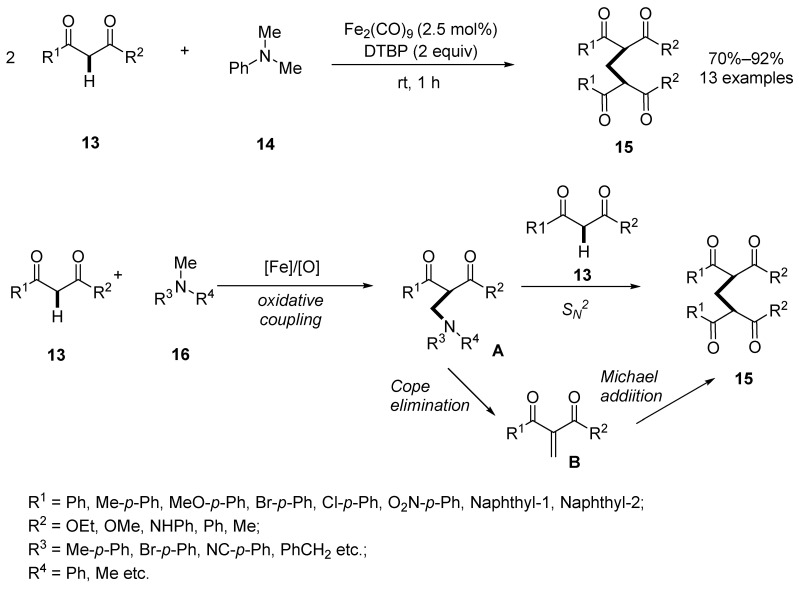
Iron-catalyzed synthesis of bis 1,3-dicarbonyl compounds.

The following year, they achieved the dialkylation of the methylene part of N,N-dimethylaniline (Scheme 6) [32]. In this reaction, under the presence of Fe_2_(CO)_9_, 1,3-dicarbonyl compounds **13** and N,N-dimethylaniline **14** could be transformed into bis-1,3-dicarbonyl compounds linked by a methylene bridge **15**. The bis-1,3-dicarbonyl products further reacted with hydrazine and ammonium acetate, respectively, yielding pyrazoles and substituted 1,4-dihydropyridines. Other N-methylamine derivatives also served as suitable substrates, but N,N-dimethylaniline derivatives with electron-withdrawing groups introduced at the para position on the benzene ring **16** resulted in lower conversion and yield rates, and aliphatic tertiary amines did not constitute an effective source of the methylene group. Additionally, the authors proposed a possible reaction pathway for forming the methylene-bridged bis-1,3-dicarbonyl products.

**Scheme 7:**
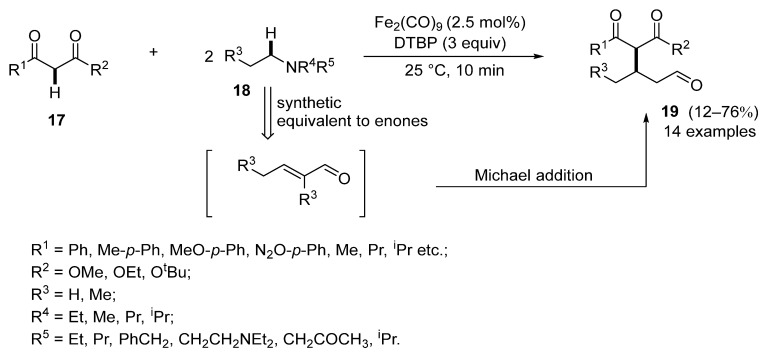
Iron-catalyzed oxidation of tertiary amines to β-1,3-dicarbonyl aldehyde.

In 2011, the same group reported the CDC reaction between two molecules of N-alkyl tertiary amine **18** and 1,3-dicarbonyl **17** in the Fe_2_(CO)_9_/TBHP system (Scheme 7) [33]. The reaction was completed within 10 min at room temperature and yielded various β-1,3-dicarbonyl aldehyde compounds. The two tertiary amine molecules initially condensed in the presence of TBHP, then the key intermediate α,β-unsaturated aldehyde underwent a Michael addition reaction, producing the final product **19**.

**Scheme 8:**
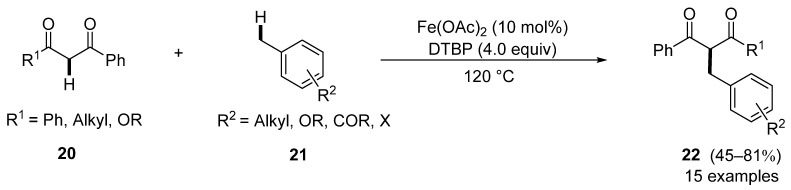
Iron-catalyzed CDC of simple toluene derivatives with 1,3-dicarbonyl compounds.

In 2012, Li group applied the iron catalyst to the CDC reaction of toluene derivatives **21** with 1,3-dicarbonyl compounds **20** (Scheme 8) [34], which exhibited good functional group tolerance. Mechanistic studies suggested that the redox from Fe^2+^ to Fe^3+^ played a key role in the formation of the C–C bond, with Fe^3+^ acting as a Lewis acid to activate the dicarbonyl substrate. The process involved the reductive homolysis of the peroxide’s O-O bond and the oxidation of carbon radicals to oxonium intermediates, confirming the role of Fe^3+^ in capturing benzyl hydrogen from the toluene derivatives.

**Scheme 9:**
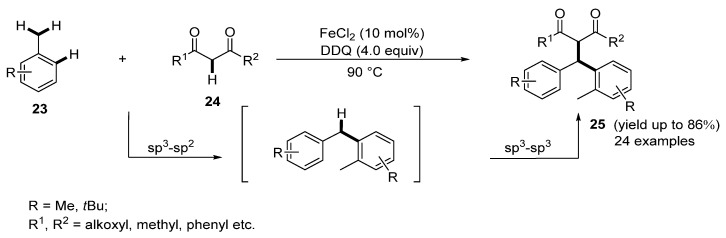
Fe-catalyzed tandem cross dehydrogenation coupling.

In 2014, Song group reported the use of DDQ as the oxidant in an iron-catalyzed dehydrogenation arylation of toluene derivatives **23** with 1,3-dicarbonyl compounds **24** through a one-pot method (Scheme 9) [35]. This scheme enabled the transformation of a new C(sp^3^)-C(sp^2^) bond and a new C(sp^3^)-C(sp^3^) bond. The tandem CDC reaction involving two C-H activations possibly starts with a Friedel-Crafts reaction, where two molecules of arylmethane self-couple under mild oxidative conditions to form a diarylmethylene intermediate, which then undergoes a CDC reaction with 1,3-dicarbonyl compounds to produce the final products **25**.

**Scheme 10:**
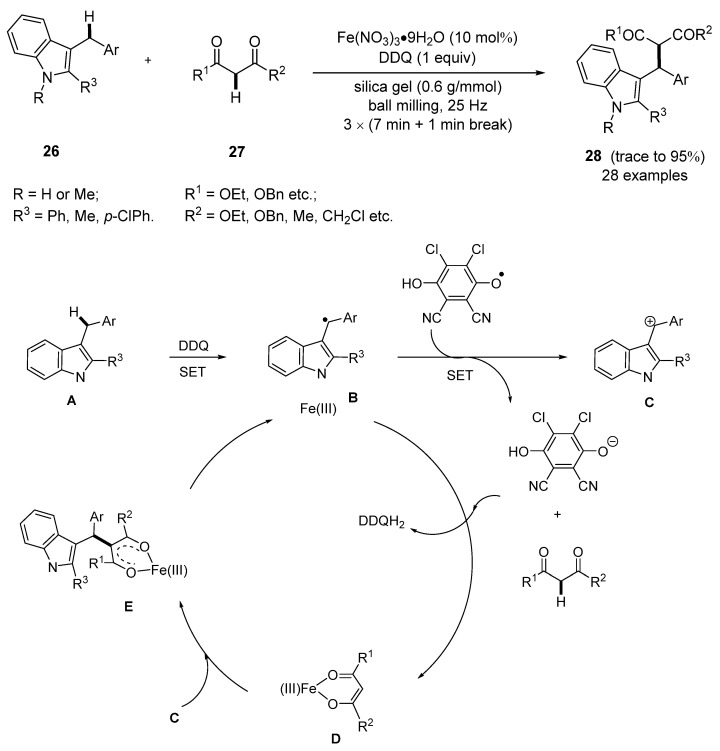
Solvent-free CDC of 3-benzylindole and methylene/indole catalyzed by Fe(III).

In 2016, Su and colleagues described a Fe(III) catalyzed CDC reaction of 3-benzylindoles **26** with methylene compounds bearing acidic protons **27** under room temperature conditions and high-speed ball-milling (HSBM) (Scheme 10) [36]. This method could achieve moderate to high yields of 3-aryl methylindole derivatives within 21 min, and the reaction conditions were also suitable for cases where 1,3-dicarbonyl compounds were substituted by indole derivatives. The reaction likely involved a free radical mechanism, initiated by two SET processes with DDQ forming the benzyl carbocation. This catalytic scheme indicates that HSBM has the potential to induce high catalytic activity of iron salt.

**Scheme 11:**
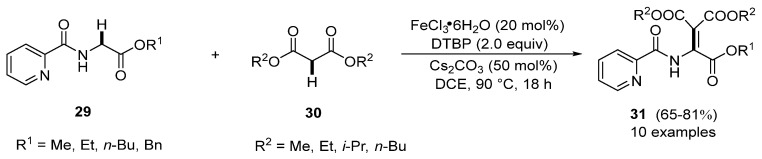
Iron-catalyzed CDC reaction of N-acylglycinate and malonate.

That same year, Yu et al. described iron catalysis for the direct formation of C-C double bonds between two C(sp^3^)-H bonds (Scheme 11) [37]. Under mild conditions, N-acylglycinates **29** reacted with malonates **30** to yield amide-acrylate esters with moderate to good yields.

**Scheme 12:**
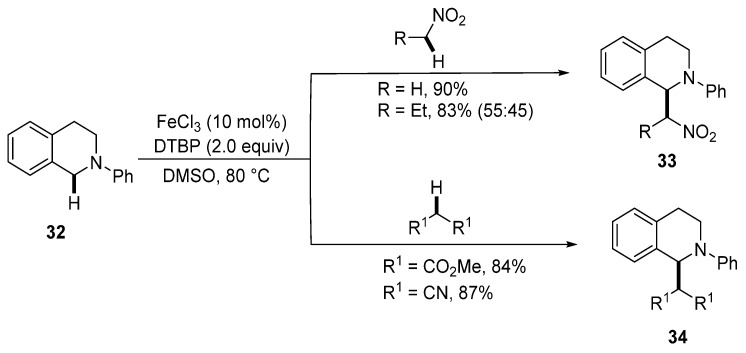
The iron-catalyzed CDC reaction of tetrahydroisoquinoline with nitroalkane and activated methylene.

Hayashi and Shirakawa reported in 2011 the FeCl_3_/DTBP catalyzed CDC reaction of tetrahydroisoquinoline **32** with nitroalkane and 1,3-dicarbonyl compounds to form aryl methylamine, α-nitro alkylamine **33**, and 2-(aminomethyl)-1,3-dicarbonyl compounds **34** (Scheme 12) [38], achieving high yields of 1-nitroalkyl substituted tetrahydroisoquinolines.

**Scheme 13:**
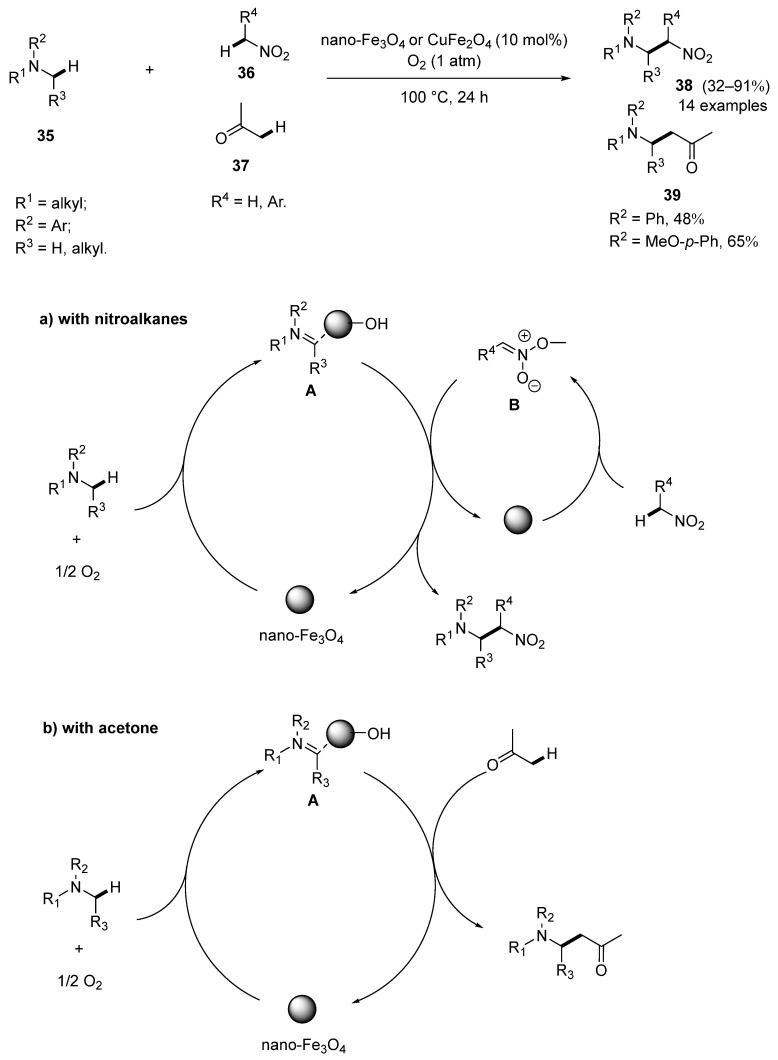
CDC catalyzed by iron nanoparticles.

In addition to the aforementioned iron halides and carbonyl iron compounds, Li’s group reported that nanoparticles based on iron oxides, such as Fe_3_O_4_ and CuFe_2_O_4_, can also serve as catalysts for CDC reactions [39,40]. In the presence of Fe_3_O_4_ nanoparticles, N-arylated analogs of tetrahydroisoquinoline **35** reacting with nitroalkanes **36** can yield alkylated products **38** (Scheme 13). CuFe_2_O_4_ can also catalyze the CDC reaction of N-arylated analogs of tetrahydroisoquinoline with various aromatic alkynes. These nanoparticles offer significant advantages as they can be recovered using an external magnet and recycled numerous times without significant loss of activity. Through catalysis by Fe_3_O_4_, tertiary amines are oxidized in situ to form iminium cations [41,42], which coordinate with Fe_3_O_4_ nanoparticles, leading to the formation of intermediate **A**. Furthermore, Fe_3_O_4_ acts as a catalyst for the deprotonation of nitroalkanes to form intermediate **B** [43]. Subsequently, the coupling of intermediates **A** and **B** yields the product and regenerates the Fe_3_O_4_ nanoparticles. Moreover, intermediate **A** can also react with acetone **37** to yield the corresponding amino ketone product **39** [44].

**Scheme 14:**
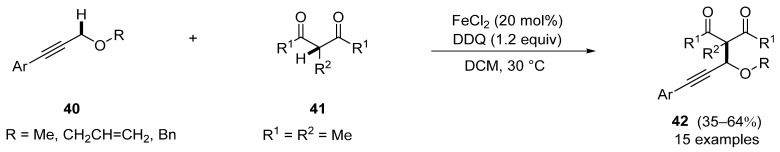
Alkylation of propargyl ether catalyzed by FeCl_2_.

Zhang and colleagues reported that in the presence of FeCl_2_ and DDQ, the oxidative dehydrogenative coupling occurs between the C(sp^3^)-H bond adjacent to the oxygen atom of a propargyl ether **40** and the active methylene C(sp^3^)-H bond of a 1,3-dicarbonyl compound **41**, yielding various propargylated β-dicarbonyl compounds **42** with moderate yields (Scheme 14) [45]. In the absence of the 1,3-dicarbonyl compound, propargyl ketones can be obtained.

**Scheme 15:**
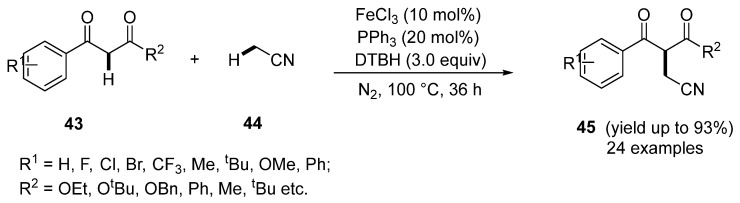
Synthesis of α-cyanomethyl-β-dicarbonyl compound by CDC reaction.

In 2016, Wu group utilized a Fe-catalyzed CDC reaction of the inert C(sp^3^)-H bond of acetonitrile **44** and the C(sp^3^)-H bond of 1,3-dicarbonyl compounds **43** to develop an efficient method for preparing α-cyanomethyl-β-dicarbonyl compounds **45**, achieving moderate to good yields of the corresponding products (Scheme 15) [46].

**Scheme 16:**
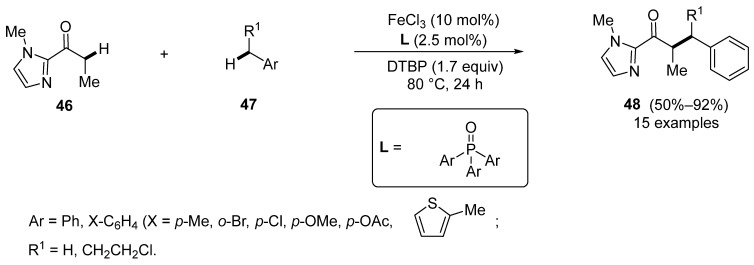
Chemoselective catalytic activation of 2-acylimidazole.

In 2018, Oshima and Yazaki described iron-catalyzed chemoselective CDC of 2-acylimidazole **46** (Scheme 16) [47], applicable to various alkyl arenes, aliphatic alkanes, acetonitriles, and functionalized 2-acylimidazoles. Additionally, this was the first time a contiguous quaternary carbon center was constructed via the CDC strategy.

**Scheme 17:**
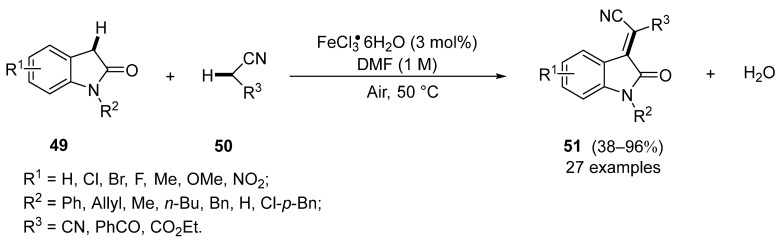
The CDC of indole-2-ones and active methylene group.

Last year, Xu’s group reported an iron-catalyzed CDC reaction of C(sp^3^)-H with C(sp^3^)-H bonds, which resulted in the formation of olefins **51** by eliminating four hydrogen protons. This method utilized green and sustainable air as the ideal oxidant, with water as the only byproduct (Scheme 17) [48].

**Scheme 18:**
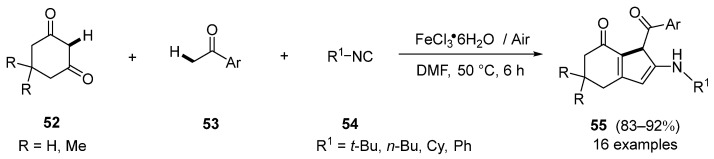
Efficient synthesis of benzofuran-4(5H)-one derivatives through CDC reaction.

In the same year, Siddiqui and colleagues developed a green and efficient reaction strategy in air, using readily available diketones **52**, acetophenone **53**, and isocyanides **54** as starting materials, employing an iron catalyst to construct biologically significant 6,7-dihydrobenzofuran-4(5H)-one derivatives **55**, achieving good to excellent yields of CDC products involving C(sp^3^)-H and C(sp^3^)-H bonds (Scheme 18) [49].

### 2.2. CDC Involving N-Methyl C(sp^3^)-H Bonds

**Scheme 19:**
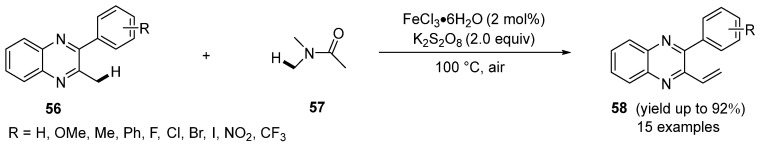
Fe-catalyzed CDC reaction generates vinyl aromatic hydrocarbons.

In 2012, Xu and colleagues utilized amides in which the N-methyl directly vinyldilated the benzyl C-H bond (Scheme 19) [50]. Various 2-methyl nitrogen heteroaromatic derivatives **56** reacted with N,N-dimethylformamide or N,N-dimethylacetamide’s N-methyl **57** to yield vinylation products **58** with good yields.

**Scheme 20:**
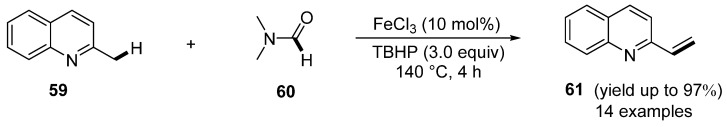
iron-catalyzed vinylation of 2-methylquinoline.

In 2013, Wang and colleagues reported an iron-catalyzed vinylation reaction of 2-methylquinoline **59** with N,N-dimethylformamide **60** in the presence of TBHP. This method synthesized various 2-vinylquinolines **61** through C(sp^3^)-H bond functionalization followed by subsequent C-N bond cleavage, using readily available 2-methylquinoline **59** as the reaction substrate, with good yields (Scheme 20) [51].

**Scheme 21:**
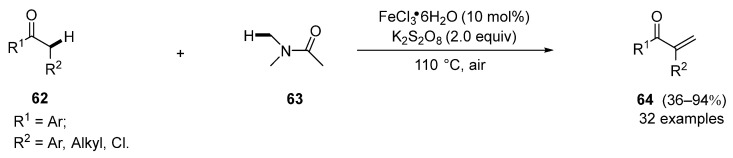
Iron-catalyzed C(sp^3^)-H activation to synthesize 2-vinylquinoline.

Li group reported in 2015 an iron-catalyzed methylenation of α-ketones using N,N-dimethylacetamide **63** as the carbon source (Scheme 21) [52]. This strategy was applicable to a variety of ketones and dicarbonyl compounds, including aromatic ketones, alkyl ketones, and enones **62**, yielding corresponding α,β-unsaturated carbonyl compounds **64** with moderate-to-good yields.

**Scheme 22:**
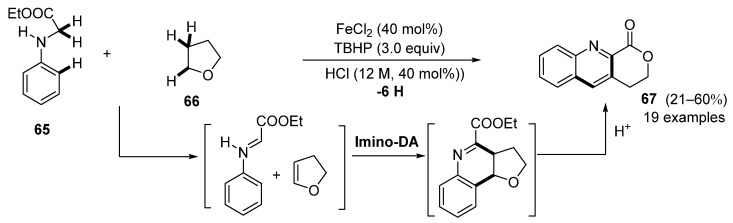
Iron-catalyzed dual-oxidative dehydrogenative (DOD) tandem annulation.

In 2015, Huo et al. reported an iron-catalyzed tandem Dual-Oxidative Dehydrogenative (DOD) and epoxidation reaction of tetrahydrofuran (THF) **66** with glycine derivatives **65** (Scheme 22) [53]. This resulted in various highly valuable quinoline-fused lactone derivatives **67** with moderate yields. The authors proposed a potential reaction mechanism involving, initially under the joint action of FeCl_2_ and TBHP, the transformation of glycine esters **65** and THF **66** into arylimines and DHF, followed by a Diels-Alder reaction producing the intermediate tetrahydroquinolizine, which, under acidic conditions, isomerized to yield the final product **67**.

**Scheme 23:**
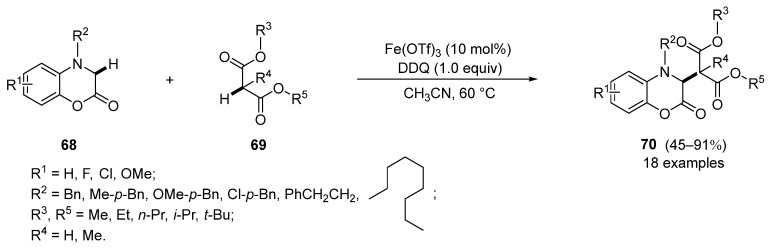
Iron-catalyzed C(sp^3^)-C(sp^3^) bond formation.

In 2016, the same group demonstrated under mild reaction conditions, through iron-catalyzed CDC reactions of malonate esters or ketones **69** with 3,4-dihydro-1,4-benzodiazepin-2-one **68**, the construction of alkyl-alkyl C-C bonds. The iron catalyst could be recycled and retained its catalytic activity without reduction (Scheme 23) [54].

**Scheme 24:**
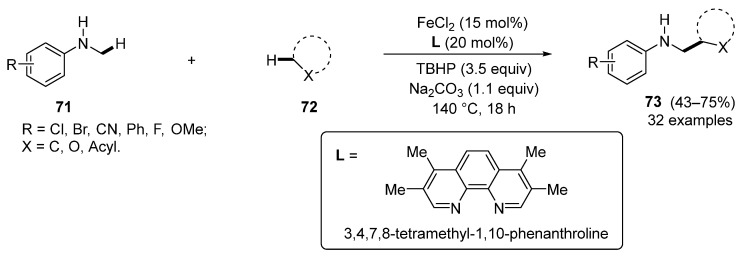
Iron catalyzes the CDC between C(sp^3^)-H and C(sp^3^)-H bonds through a radical process.

In 2019, Cai group first developed a region-selective ligand-promoted CDC reaction between unactivated C(sp^3^)-H and C(sp^3^)-H bonds (Scheme 24) [55]. Various types of C(sp^3^)-H bond, including cycloalkanes, cyclic ethers, and toluene derivatives without any directing groups **72**, could be used as coupling substrates. The ligand 3,4,7,8-tetramethyl-1,10-phenanthroline acted as an activator of the catalyst promoting the reaction, with the anion bound to iron playing a crucial role in the catalysis. The reaction could proceed through a free radical pathway. Iron played a significant role in electron transfer, with TBHP acting as both oxidant and radical initiator.

## 3. Iron-Catalyzed C(sp^3^)-H with C(sp^2^)-H Cross-Dehydrogenative Coupling

### 3.1. CDC Involving Aryl C(sp^2^)-H Bonds

#### 3.1.1. Activation of Non-Heteroatom Adjacent C(sp^3^)-H Bonds

**Scheme 25:**
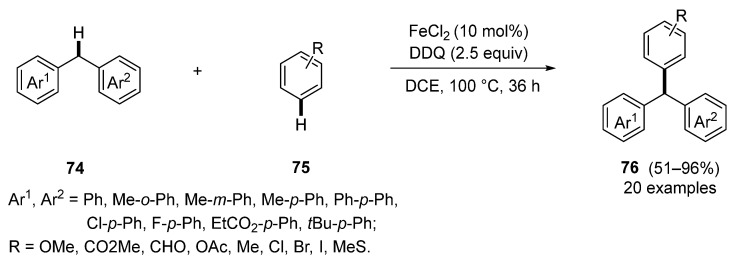
Iron catalyzes CDA of aryl C(sp^2^)-H bonds and aromatic C(sp^3^)-H bonds.

In 2009, Professor Shi and his colleagues first reported an iron-catalyzed intermolecular benzyl C(sp^3^)-H bond **74** with aromatic **75** CDC reaction (Scheme 25) [56]. Using FeCl_2_ as the catalyst and DDQ as the oxidant, electron-rich arenes could react with dibenzylmethane **74** to yield CDC products, with good yields. Additionally, increasing the electron density of the arenes could enhance the yield of the coupling products, consistent with their nucleophilic character, and further indicate the presence of a Friedel-Crafts alkylation process.

**Scheme 26:**
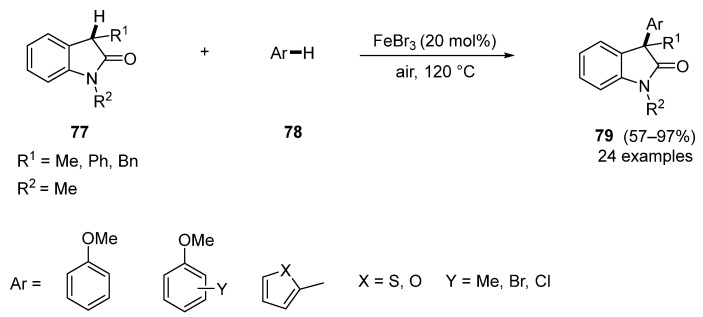
Iron catalyzes the CDC of aryl C(sp^2^)H bonds and aromatic C(sp^3^)H bonds.

In 2015, Li’s group developed an efficient FeIII-catalyzed cross dehydroarylation (CDA) of 3-substituted hydroxyindoles **77** with electron-rich aromatic and heteroaromatic compounds **78** in an air atmosphere, synthesizing 3,3′-disubstituted hydroxyindoles **79** with higher yields (Scheme 26) [57].

**Scheme 27:**
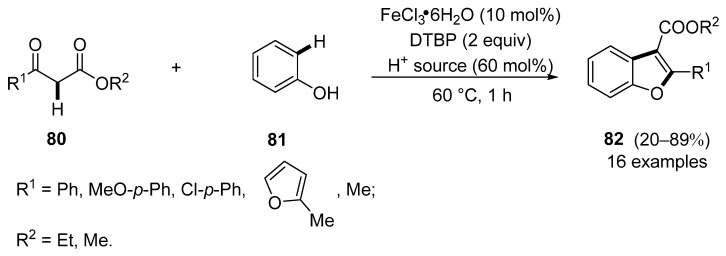
Iron-catalyzed tandem CDC and cyclization to construct polysubstituted benzofurans.

Li and colleagues reported the synthesis of polysubstituted benzofurans **82** through an iron-catalyzed tandem CDC and intramolecular condensation cyclization reaction (Scheme 27) [58]. Experiments showed that water and various alcohols and protic acids could promote this reaction, and when 4 Å molecular sieves were added to the reaction system, the reaction was terminated. Isotope effects indicated the rate-determining step was not the aromatic compound’s C-H bond cleavage. Moreover, the iron catalyst acted as a transition metal catalyst in the oxidative coupling step and as a Lewis acid in the condensation step.

**Scheme 28:**
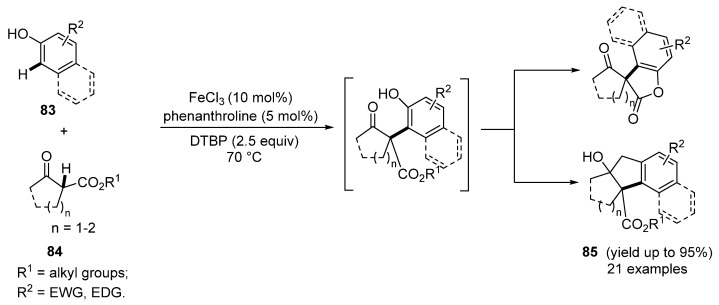
Ligand-controlled iron-catalyzed cross-dehydrogenation coupling.

In 2012, Pappo and his colleagues reported an iron-catalyzed coupling of cyclic α-substituted β-ketoesters **84** with phenols **83**, using catalytic amounts of 1,10-phenanthroline as a ligand, synthesizing polycyclic hemiacetals or polycyclic spirolactones **85** (Scheme 28) [59]. This CDC reaction exhibited chemoselectivity, regioselectivity, and stereochemistry control. Here, the 1,10-phenanthroline ligand acted as a promoter of the CDC reaction and an inhibitor of side reactions.

**Scheme 29:**
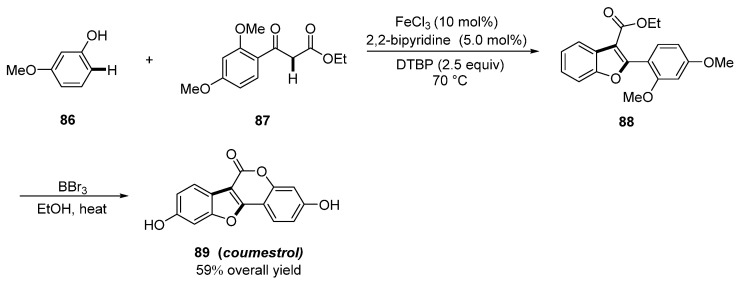
CDC based on iron catalysis realizes the synthesis of selective estrogen receptor modulators based on coumarin.

Additionally, Pappo and his team successfully applied this method to the total synthesis of coumarin. The reaction system of FeCl_3_ and 2,2′-bipyridine with DTBP was suitable for CDC reactions of phenols **86** with 2-benzyloxyethyl acetate derivatives **87** (Scheme 29) [60], achieving good to high yields of the corresponding benzofurans **88**. The benzofurans 88 obtained through deprotection and lactonization steps could be transformed into selective estrogen receptor modulators **89** derived from coumarin. This was also the first instance of applying the CDC reaction to total synthesis to obtain natural products.

**Scheme 30:**
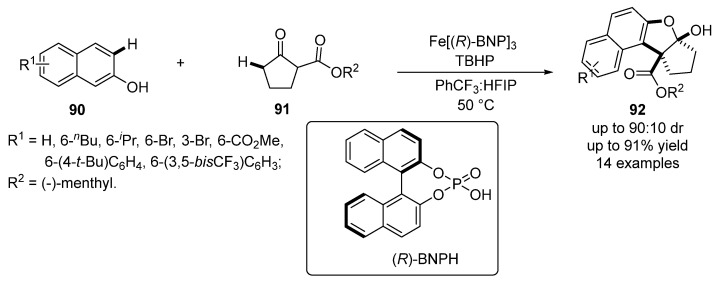
Asymmetric cross dehydrogenation coupling reaction catalyzed by iron phosphate.

In 2017, based on the CDC strategy, the same research group developed an iron phosphate-catalyzed asymmetric CDC reaction between 2-naphthols **90** and β-ketoester derivatives **91**, synthesizing polycyclic hemiacetals **92** (Scheme 30) [61]. Kinetic studies suggested that the mono iron phosphate complex acted as a stereoselectivity-inducing active redox catalyst in the carbon-carbon bond formation step, with coupling occurring between two ligands involved in a radical-anion coupling mechanism.

**Scheme 31:**
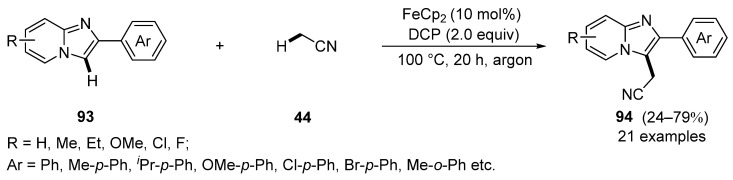
Iron-catalyzed construction of C(sp^3^)-C(sp^2^).

In 2017, a CDC reaction catalyzed by iron involving 2-aryl imidazo [1,2-a]pyridine’s C(sp^2^)-H bond **93** with acetonitrile’s C (sp^3^)-H bond **44** was realized, a strategy suitable for preparing heteroaryl acetonitriles **94**. This transformation was notable for its tolerance to a variety of functional groups and its efficiency in directly synthesizing pharmaceutical zolpidem on a heteroaryl acetonitrile base (Scheme 31) [62]. This method is expected to have significant implications for synthetic chemistry and pharmaceutical chemistry.

**Scheme 32:**
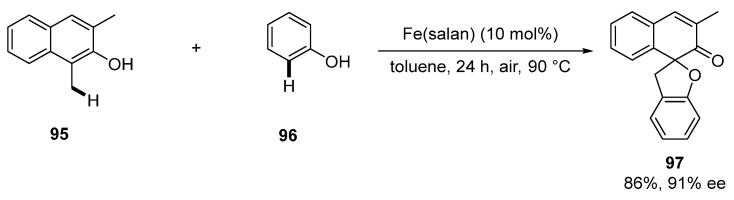
Iron-catalyzed asymmetric tandem spirocyclization.

In 2014, Katsuki and his colleagues reported an iron-catalyzed asymmetric enantioselective tandem spirocyclization reaction, employing a chiral Fe(salan) complex as the catalyst and air as the oxidant, with phenols **96** and naphthols **95** as substrates. They achieved an isolated yield of 86% and an enantiomeric excess (ee) value of 91%, producing spirocyclic (2H) dihydrobenzofurans **97** (Scheme 32) [63]. This iron-catalyzed oxidative o-quinone methide (o-QM) formation/Michael addition/asymmetric de-aromatization tandem strategy provided an efficient method for synthesizing spirocyclic compounds.

**Scheme 33:**
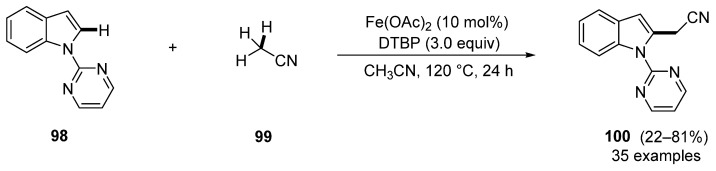
Fe(II) catalyzed CDC of indoles/pyrrole with acetonitriles.

In 2018, Guo and co-workers reported a method for coupling indoles and pyrrole **98** C(sp^2^)-H with acetonitrile derivatives **99** C(sp^3^)-H using Fe(II) as the catalyst, achieving moderate to good yields of CDC products **100** (Scheme 33) [64]. This reaction exhibited good functional group compatibility, a broad substrate range, and good regioselectivity. This reaction strategy was also used for the C-H activation of bioactive compounds, achieving high transformation yields.

**Scheme 34:**
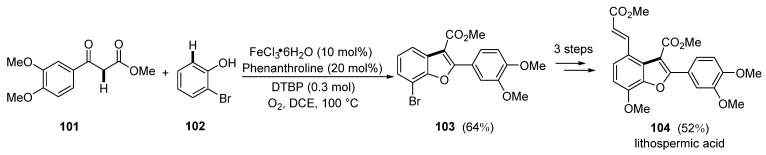
Fe-catalyzed CDC reaction to synthesize lithospermic acid.

In 2018, Professor Xuan and his colleagues synthesized lithospermic acid **104** through a CDC reaction between o-bromophenol **102** and β-ketoesters **101**, where double C-H activation was a key step. Here, the iron-catalyzed CDC reaction was employed for the construction of the benzofuran skeleton **103**, and Pd-catalyzed ester-directed C-H alkenylation effectively constructed the dihydrobenzofuran ester portion (Scheme 34) [65].

**Scheme 35:**
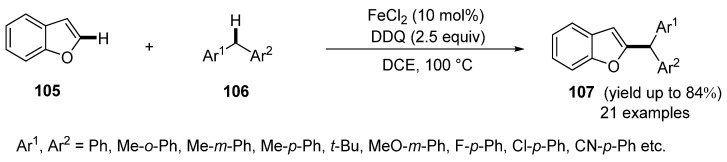
FeCl_2_-catalyzed CDC of diarylmethane with benzofuran.

In 2021, Shen’s team reported an iron-catalyzed CDC of benzofuran **105** with unactivated diarylmethane **106** (Scheme 35) [66]. Starting from readily available compounds using Fe(II)/DDQ, benzyl groups were introduced region-selectively to the C_2_-position of the benzofuran skeleton. This was the first example of a highly regioselective C_2_-benzylation of benzofuran with unactivated diarylmethane via a CDC reaction.

**Scheme 36:**
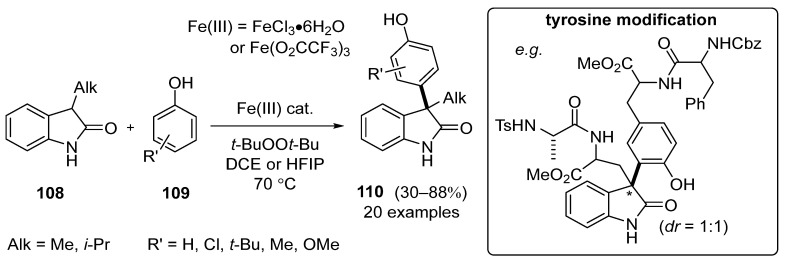
Iron-catalyzed oxidative cross-coupling for oxindole functionalization.

In 2021, Pappo’s research group published iron-catalyzed oxidative cross-coupling of phenols **109** and tyrosine derivatives with 3-Alkyloxindoles **108** (Scheme 36) [67]. This study describes an innovative iron-catalyzed oxidative cross-coupling reaction between phenols and 3-alkyloxindole derivatives. Using FeCl_3_ as the catalyst and t-BuOOt-Bu as the oxidant in 1,2-dichloroethane at 70 °C, this method selectively produces 3-alkyl-3-(hydroxyaryl)oxindole compounds. The scope of this approach was demonstrated with various substituted phenols, naphthols, and tyrosine derivatives reacting with 3-alkyloxindoles. Furthermore, this methodology was adapted for conjugating tyrosine-containing short peptides with oxindolylalanine (Oia) derivatives.

#### 3.1.2. Activation of C(sp^3^)-H Bonds Adjacent to Heteroatoms

**Scheme 37:**
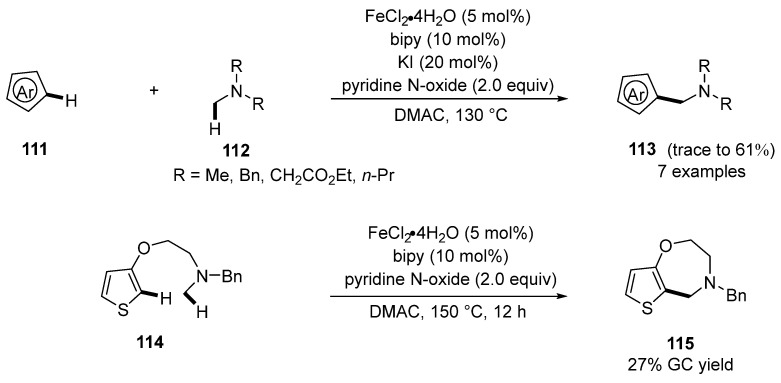
Iron-catalyzed cross dehydrogenation coupling of heteroarene with methylamine.

In 2009, Itami and his colleagues reported a new iron-catalyzed CDC system applicable for rich-electron heteroarene C-H bonds **111** and N-methyl C-H bonds **112** (Scheme 37) [68]. They also applied this reaction scheme to intramolecular oxidative coupling, forming a phenanthridine dibenzazepine structure **115**. The transformation primarily proceeded through hydrogen atom abstraction from the N-methyl C-H bond to form an iminium ion intermediate, allowing selective activation at the N-methyl C-H bond.

**Scheme 38:**
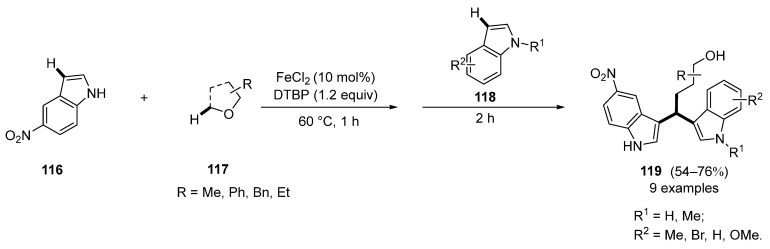
1,1-bis-indolemethane were synthesized by one-pot method.

Li and his colleagues achieved an iron-catalyzed CDC reaction of indole **116**, **118** C(sp^2^)-H and ether **117** C(sp^3^)-H bonds to form symmetrical and asymmetrical 1,1-bis-indolylmethane derivatives **119** (Scheme 38) [69]. The reaction proceeded through a tandem C-H bond oxidative coupling and C-O bond cleavage, with high efficiency. The iron catalyst acted as a transition metal catalyst in the C-C bond oxidative coupling and as a Lewis acid in the C-O bond cleavage. The introduction of the two indoles was believed to occur in separate steps involving a radical process and Friedel-Crafts alkylation.

**Scheme 39:**
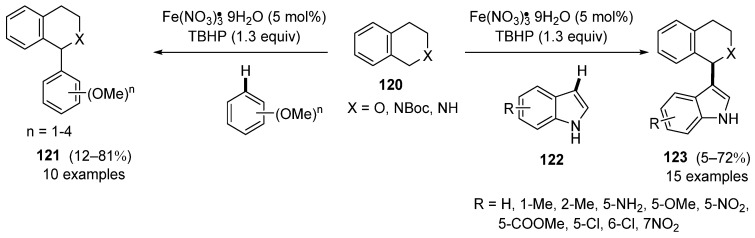
Iron-catalyzed functionalization of tetrahydroisoquinoline and isochroman.

Schnurch and his colleagues reported in 2010 an iron-catalyzed method for the oxidative indolation and arylation of N-Boc protected tetrahydroisoquinoline **120** and isochroman, using substrates such as tetrahydroisoquinoline and isochroman, and achieving good or excellent yields of the desired coupling products **121**, **123** (Scheme 39) [70].

**Scheme 40:**
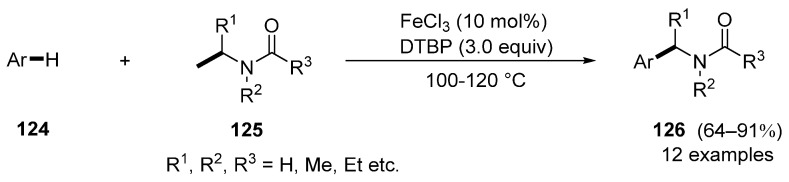
Iron-catalyzed CDC reaction of alkyl amides with aromatic hydrocarbons.

In 2011, Hiyashi and colleagues discovered that an FeCl_3_ with TBHP reaction system could oxidize alkyl amides **125** to R-(tert-butoxy) alkyl amides **126**, and the Fe(III) acting as a Lewis acid facilitated the subsequent Friedel-Crafts alkylation to proceed smoothly (Scheme 40) [71].

**Scheme 41:**
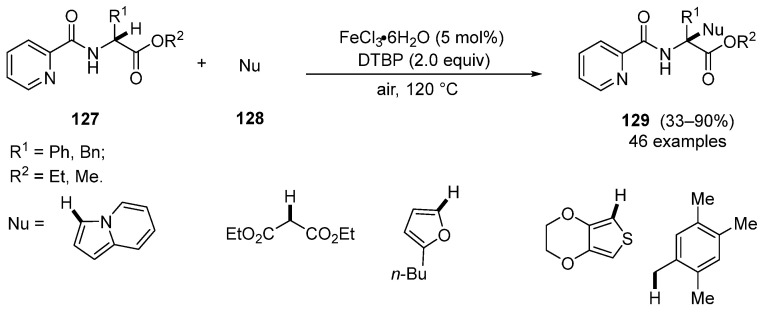
A highly efficient α-quaternary-α-amino acid derivative pathway catalyzed by iron.

In 2013, You and his colleagues developed a coordination activation strategy, whereby an iron(III) catalyzed CDC reaction of α-C(sp^3^)-H bonds in α-amino acid esters **127** substituted with N-(pyridin-2-ylcarbonyl) and indole nucleophiles **128** was used to construct a series of high-yield α-quaternary-α-amino acid derivatives **129** (Scheme 41) [72]. This reaction system displayed a broad substrate scope for both α-amino acids and nucleophiles and good functional group tolerance. The 2-pyridylcarbonyl group, due to its strong coordinating and good elimination capacity, emerged as an ideal substituent for the N-protecting group.

**Scheme 42:**
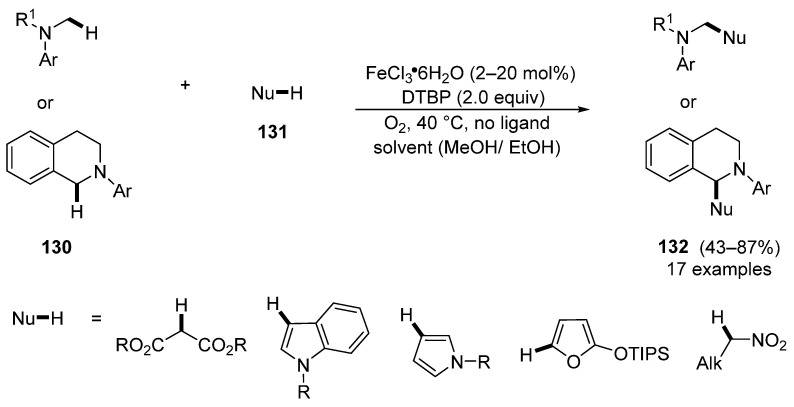
Iron-catalyzed C-H functionalization of N,N-dialkylanilines.

Doyle and colleagues reported an iron chloride-catalyzed activation of the N,N-dialkylaniline **130** C-H bond adjacent to the nitrogen atom under an oxygen atmosphere as the oxidant (Scheme 42) [73]. Nucleophiles **131** such as methoxysilane furan and nitroalkane participated effectively in this reaction process.

**Scheme 43:**
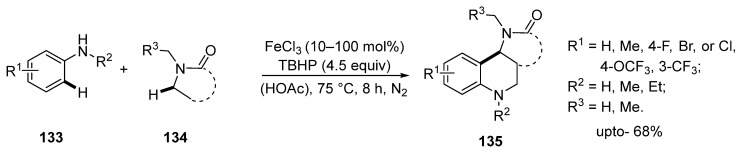
FeCl_3_ catalyzed one-pot synthesis of ring-fused tetrahydroquinoline derivatives.

Bao and his colleagues reported in 2013 a FeCl_3_-catalyzed reaction of arylamines **133** with N-substituted lactams **134**, yielding tetrahydroquinoline ring-fused compounds **135** [74] (Scheme 43). The proposed mechanism involved the formation of two iminium ions by single-electron transfer from the N-substituted lactams **134**, with the subsequent Friedel–Crafts-type reaction and nucleophilic addition with arylamines **133** forming an unclosed intermediate with two N-substituted lactams, which finally, through C–N bond cleavage, transformed into the target compound **135** [75].

**Scheme 44:**
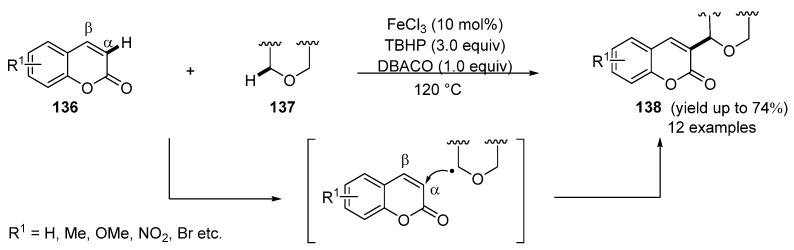
Regioselective cross dehydrogenation coupling of coumarin and flavonoids with ethers.

Coumarin and flavonoid derivatives are very valuable precursors in drug synthesis. In 2015, Ge Haibo and colleagues developed a region-selective and atom-economic CDC reaction of coumarin and flavonoids **136** with different ethers **137**, yielding two new types of ether-substituted derivatives **138**. In this reaction, ether substituents were introduced at the electron-rich α-position of coumarin and the β-position of flavonoids (Scheme 44) [76].

**Scheme 45:**
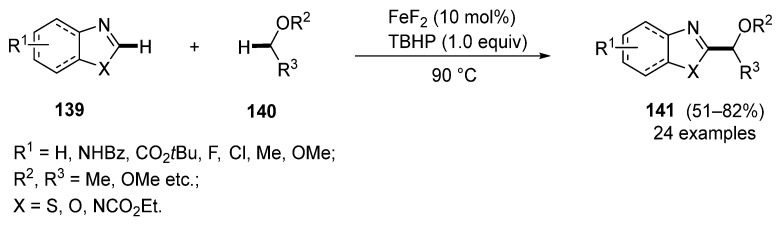
Iron-catalyzed cross dehydrogenation coupling reaction of ether and pyrrole.

That same year, Correa and his team reported an FeF_2_-catalyzed direct α-arylation of azoles **139** with ethers **140** (Scheme 45) [77], enabling practical oxidative methods that facilitated effective C-2 alkylation of various (benzo)azoles, yielding heterocycles **141** of medicinal value. The authors also discussed the reaction mechanism supported by DFT calculations, highlighting the important redox role of FeF_2_ in assisting the oxidative cleavage of the hybrid and oxidation of the carbon radical to an oxocarbenium intermediate.

**Scheme 46:**
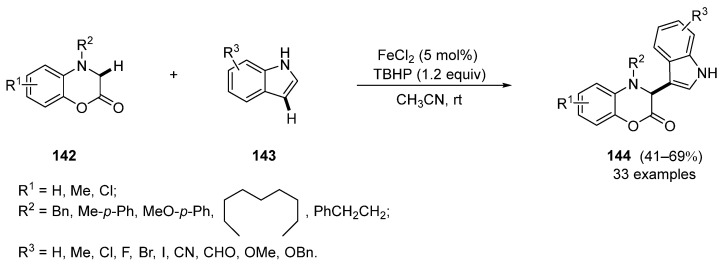
Iron-catalyzed activation of the C(sp^3^) bond.

In 2016, Huo and others developing an iron-catalyzed activation of the C(sp^3^)-H bond of 3,4-dihydro-1,4-benzodiazepin-2-one derivatives **142**. Conducted under mild reaction conditions, this method constructed alkyl-aryl C(sp^3^)-C(sp^2^) bonds and could participate in the total synthesis of the natural product cephalosporin on a kilogram scale (Scheme 46) [78]. The key steps involved a radical process, and the authors proposed a potential mechanism involving electronic oxidation participation.

**Scheme 47:**
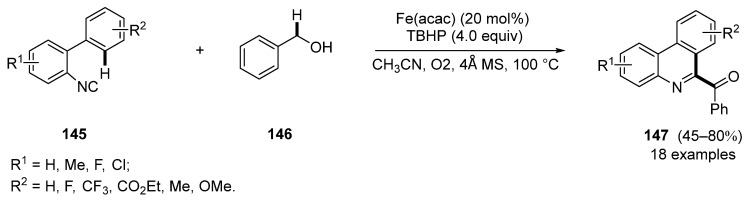
6-Aroyl phenanthridines were synthesized by Fe-catalyzed CDC reaction.

Ding Qiuping and colleagues reported an Fe-catalyzed oxidative radical cyclization reaction of 2-isocyanobiaryl **145** C(sp^2^)-H bonds with benzyl alcohol **146** at the benzylic C(sp^3^)-H bond, synthesizing 6-aroyl phenanthridine derivatives **147** [79] (Scheme 47). The strategy involved pathways such as oxidative, intermolecular radical addition, intramolecular radical cyclization, deprotonation, and single-electron transfer in a one-pot tandem reaction sequence.

**Scheme 48:**
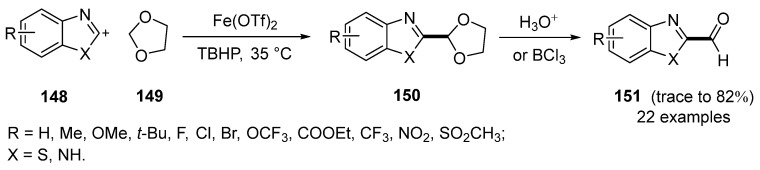

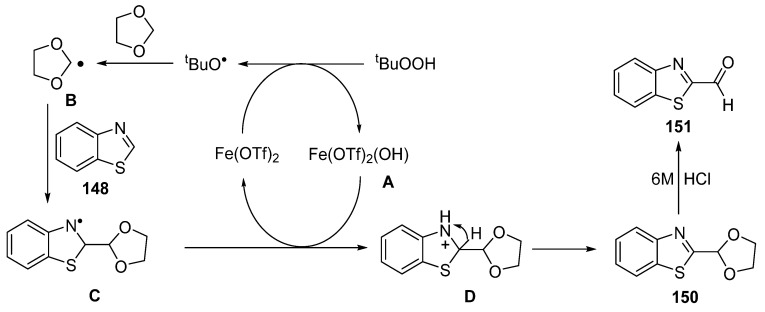
Iron-catalyzed tandem oxidative coupling and acetal hydrolysis reaction to prepare formylated benzothiazoles and isoquinolines.

In 2021, Wang and Xiong et al. reported an iron-catalyzed tandem oxidative coupling andacetal hydrolysis reaction to prepare formylated benzothiazoles **151** and isoquinolinest. The process begins with a single-electron transfer (SET) reduction of TBHP, which simultaneously oxidizes Fe(OTf)_2_ to Fe(OTf)_2_OH and generates a tert-butyl oxygen radical (t-BuO·). This radical abstracts hydrogen atoms from 1,3-dioxolane **149** to form alkyl radical **B**. The newly formed radical **B** subsequently attacks the benzothiazole-derived intermediate **C**, leading to the formation of cationic intermediate **D** through oxidation. Proton release and re-aromatization of D yield product **150**. In the final step, **150** undergoes acid hydrolysis to afford the desired product **151**. Moreover, Fe(OTf)_2_ plays a crucial role in completing the catalytic cycle by facilitating both the homolytic cleavage of TBHP and the oxidation of carbon radical **C** to cation **D** [80] (Scheme 48).

### 3.2. CDC Involving Alkene C(sp^2^)-H Bonds

**Scheme 49:**
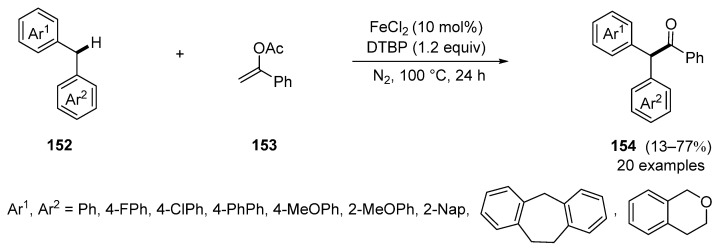
Direct functionalization of benzyl C-H.

In 2009, Shi’s research group achieved an iron-catalyzed CDC reaction of ethyl acrylate **153** with benzyl **152** C–H bonds (Scheme 49) [81], obtaining the coupling products **154** with excellent yields. The reaction system demonstrated good functional group tolerance for functionalized diarylmethanes and was also applicable to the Mizoroki–Heck type reaction of styrene with dibenzylmethane. The authors considered that the reaction could proceed through two pathways, both initiated by SET, involving either a radical or a cationic pathway.

**Scheme 50:**
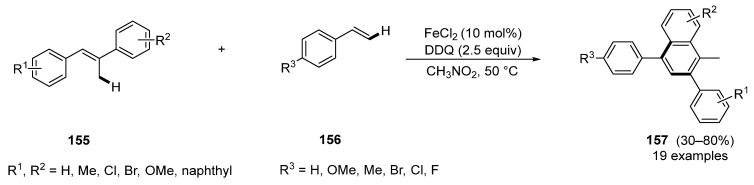
CDC reaction of terminal allyl C(sp^3^)-H with styrene C(sp^2^)-H.

In 2012, Deng and colleagues reported a novel tandem iron-catalyzed CDC and benzannulation reaction of terminal allylic C(sp^3^)-H with styrene C(sp^3^)-H, starting from simple 1,2-arylpropenes **155** and styrene **156** to synthesize polysubstituted naphthalene derivatives **157** with significant biological and synthetic importance, achieving moderate to good yields (Scheme 50) [82].

**Scheme 51:**
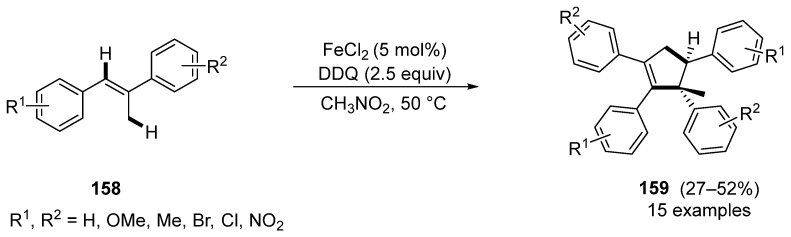
The substituted cyclopentenes were synthesized by tandem CDC and cyclization.

That same year, they used (E)-1,2-diarylprop-1-ene **158** in the same reaction system to form highly stereo-selective trans-substituted 1,2,3,4-tetraphenylcyclopentenes **159** through tandem CDC and cyclization, representing the first example of constructing cyclopentene derivatives through C(sp^3^)-C(sp^2^) bond oxidative coupling (Scheme 51) [83].

**Scheme 52:**
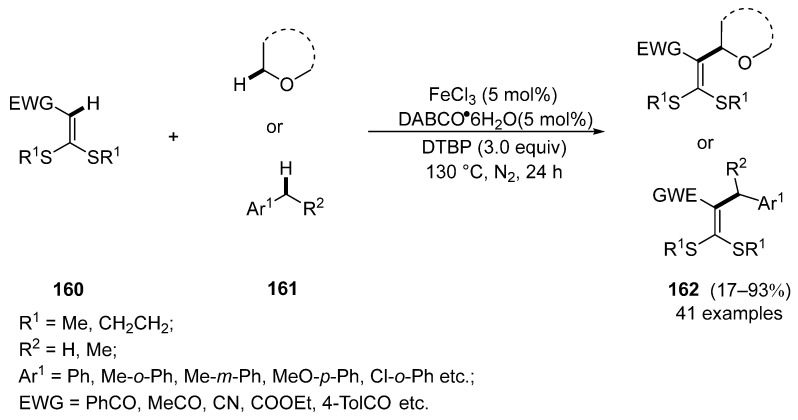
Iron-mediated oxidative C–H alkylation of S,S-functionalized internal olefins.

Under catalytic amounts of FeCl_3_, Yu and colleagues using peroxide DTBP as the oxidant and DABCO·6H_2_O as an additive, employing simple ethers or benzyl derivatives **161** as coupling agents, the effective alkylation of S,S-Functionalized Internal Olefins **160** was achieved, yielding tetrasubstituted olefinic compounds that could be used to prepare trisubstituted NH-pyrazoles and isoxazoles **162** (Scheme 52) [84].

**Scheme 53:**
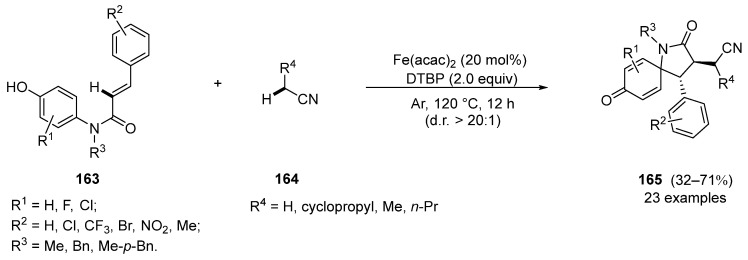
Iron-catalyzed cyanoalkylation/free radical dearomatization of N-phenylcinnamamide.

In 2017, Zhu et al. developed an iron-catalyzed tandem cyanoalkylation and free radical dearomatization spirocyclization of N-phenylcinnamamide **163**, initiated by the cyanoalkylation of alkenes, yielding coupled products **165** with good regioselectivity and diasteroselectivity (Scheme 53) [85].

## 4. Iron-Catalyzed C(sp^3^)-H with C(sp)-H Cross-Dehydrogenative Coupling

**Scheme 54:**
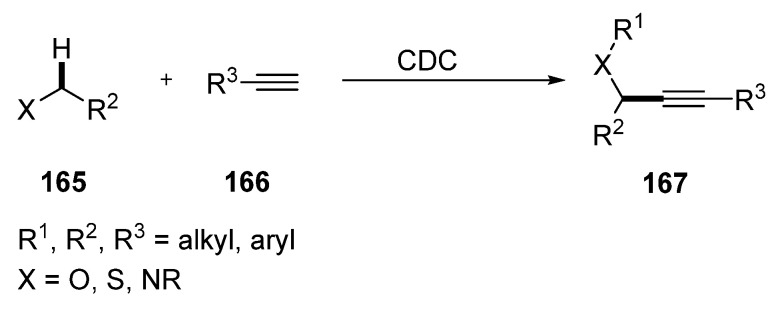
72 Cross-dehydrocoupling reaction of C(sp^3^)-H with C(sp)-H.

Compared to iron-catalyzed CDC reactions between C(sp^3^)-H and C(sp^3^)-H/C(sp^2^)-H, there are few examples of iron-catalyzed C(sp^3^)-H with C(sp)-H CDC reactions, and such reactions typically involve C(sp^3^)-H bonds adjacent to N, O, or S atoms **165** (Scheme 54) [4].

**Scheme 55:**
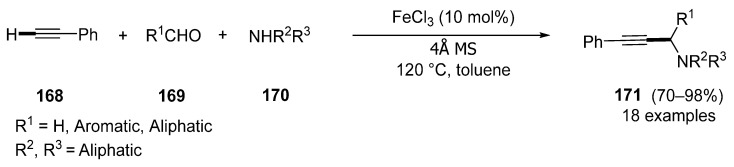
Iron-catalyzed coupling reaction of aldehydes, terminal alkynes, and amines.

Wang Lei and his colleagues reported a ligand-free FeCl_3_-catalyzed three-component coupling reaction of aldehydes **169**, terminal alkynes **168**, and amines **170** to construct propargylamines **171** (Scheme 55) [86].

**Scheme 56:**
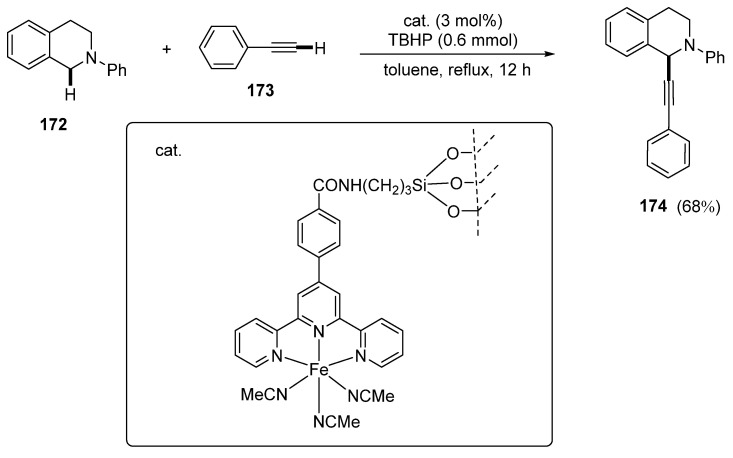
SBA-15-supported iron pyridine-catalyzed carbon-carbon bond coupling.

In 2010, Professor Che reported the use of an SBA-15-supported tris(pyridine) iron complex to efficiently catalyze the CDC reaction of tertiary amines **172** with carbon nucleophiles (Scheme 56) [87]. The tertiary amine C(sp^3^)-H bonds could be coupled not only with carbon nucleophiles having C(sp^2^)-H bonds but also with those having C(sp)-H bonds **173**. Moreover, the supported pyridine ligands could be filtered, recovered, and reused.

**Scheme 57:**
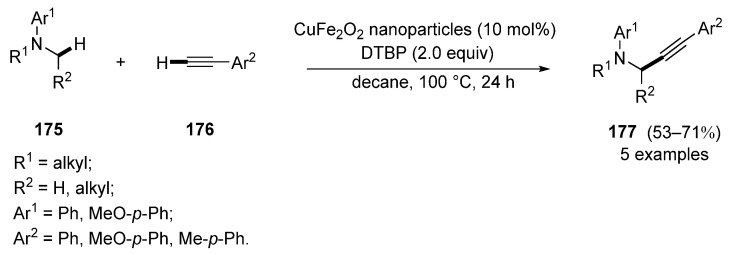
CuFe_2_O_4_ catlyzed CDC of aryl amines with acetylenes.

Professor Li and his colleagues in 2013 reported a CDC reaction catalyzed by nanoparticle CuFe_2_O_4_, facilitating C(sp^3^)-C(sp) coupling (Scheme 57) [40]. The authors believed that both iron and copper were effectively involved in the CDC of C(sp^3^)-H bonds, with the copper centers coordinating with acetylenes **176** to form acetylide copper, activating C(sp)-H for coupling with a C(sp^3^) center, while DDQ oxidation of tertiary amines **175** generated iminium ions that coordinated to adjacent Fe or Cu atoms on the nanoparticles, ultimately coupling both to yield the target product **177** and regenerating the CuFe_2_O_4_ nanoparticles to complete the catalytic cycle.

**Scheme 58:**
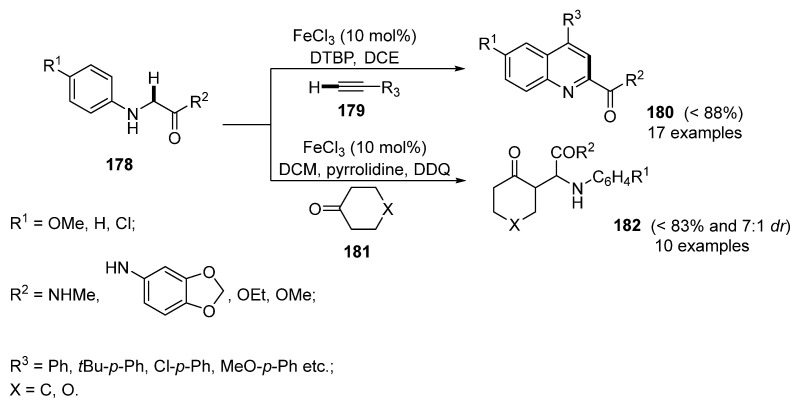
Iron catalyzes the CDC reaction of glycine derivatives.

In 2012, Professor Hu and his colleagues reported the FeCl_3_-catalyzed CDC reaction of phenylglycine derivatives **178** with substituted acetylenes **179** to construct quinolines **180** with high yields (Scheme 58) [88]. Additionally, when using pyrrolidine as an organic catalyst, the reaction of phenylglycine derivatives **178** with cyclic ketones **181** also yielded the corresponding coupling products **182**. The inclusion of radical scavengers in the reaction system resulted in a drastic decrease in the yield of the target products, suggesting that both reactions likely involved the generation of radicals.

**Scheme 59:**
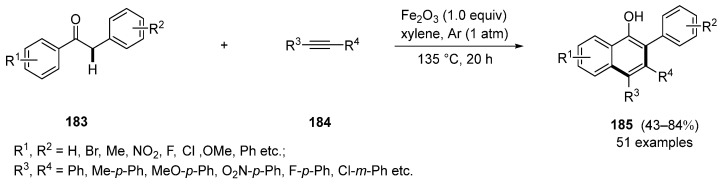
β-aryl-α-naphthol was synthesized by iron-catalyzed tandem CDC reaction.

In 2020, Wang’s group reported an iron-promoted tandem CDC reaction of C(sp^3^)-H with C(sp)-H and C(sp^2^)-H with C(sp)-H, using deoxybenzoin **183** and alkynes **184** as starting materials to develop a synthetic strategy for β-aryl-α-naphthol **185** (Scheme 59) [89]. This strategy features inexpensive and readily available substrates, a wide substrate scope, simple operation, and high regioselectivity.

**Scheme 60:**
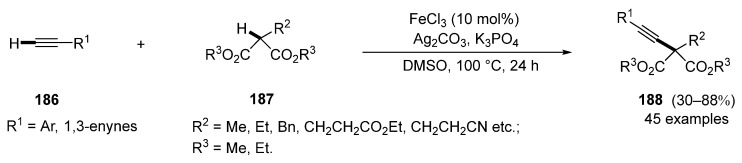
Iron-catalyzed CDC reaction of terminal alkynes with 1,3-diesters.

Reactivity and site selectivity in the cleavage of C(sp^3^)-H bonds continue to pose ongoing challenges. In 2021, Hu’s research group provided a CDC scheme involving an iron catalysis/silver-mediated reaction of 1,3-dicarbonyl compounds **187** with terminal alkynes **186** achieving high selectivity in tertiary alkylation, with aromatic acetylenes and 1,3-enynes serving as viable substrates (Scheme 60) [90].

## 5. General Trends

Recent developments in iron-catalyzed CDC reactions have demonstrated their applicability to a wide range of C(sp^3^)-H, C(sp^2^)-H, and C(sp)-H bonds. These reactions exhibit excellent functional group tolerance and compatibility with complex substrates, including 1,3-dicarbonyl compounds, benzylic derivatives, ethers, and nitrogen-containing heterocycles. However, expanding the substrate scope to include more diverse and challenging compounds remains highly desirable, as it would further enhance the applicability and impact of this methodology in synthetic chemistry. Advanced catalytic systems have shown remarkable selectivity, particularly in regioselective and stereoselective transformations. For instance, ligand-modulated iron-catalyzed CDC reactions have enabled the highly selective synthesis of chiral polycyclic compounds. Iron-catalyzed CDC reactions are increasingly being performed under milder conditions. High-speed ball milling (HSBM) and solvent-free systems have successfully facilitated reactions at room temperature with high yields, reducing reliance on high-temperature and high-pressure setups while minimizing solvent usage. These reactions commonly proceed via radical mechanisms or single-electron transfer (SET) pathways. This versatility allows them to accommodate a broader range of transformations, such as reactions involving enolate intermediates or carbene species. The emergence of iron-based nanoparticles and functionalized catalysts has expanded the scope of CDC reactions. For example, magnetic nanocatalysts (e.g., Fe_3_O_4_ and CuFe_2_O_4_) have shown high catalytic activity and can be recovered and reused using external magnetic fields, enhancing the cost-efficiency of the catalytic process.

## 6. Conclusions

The development of iron-catalyzed CDC reactions marks a transformative progress in the synthesis of complex molecules, aligning with the goals of sustainable and green chemistry. Iron’s role as a catalyst is especially valuable due to its low cost, abundance, and low environmental impact compared to traditional precious metal catalysts. These reactions have proven versatile in creating a wide array of chemical bonds and structures under mild, eco-friendly conditions. As the field grows, future research is expected to further refine these methods, improving their efficiency, scope, and applicative value in both laboratory and industrial settings. Enhancing the understanding of iron’s catalytic mechanisms will also open new avenues for innovation in CDC and other types of organic reactions, solidifying iron’s status as a cornerstone in sustainable chemical practices.
